# An Overview of Meningococcal Disease's Recent Diagnostic and Treatment Model

**DOI:** 10.7759/cureus.48509

**Published:** 2023-11-08

**Authors:** Deepali Chhabria, Ashish Anjankar

**Affiliations:** 1 Medicine, Jawaharlal Nehru Medical College, Datta Meghe Institute of Higher Education and Research, Wardha, IND; 2 Biochemistry, Jawaharlal Nehru Medical College, Datta Meghe Institute of Higher Education and Research, Wardha, IND

**Keywords:** meningococcal vaccination, vaccines development, neisseria meningitidis, invasive meningococcal disease, meningococcal disease

## Abstract

In healthy people, *Neisseria meningitidis* (the meningococcus) is a typical component of the nasopharyngeal microbiome, but in those who are susceptible, it can cause septicemia and meningitis. This section gives a general overview of the meningococcus types and the sickness induced by *N. meningitidis.* Evaluate genes for phase-changeable adhesions, virulence factors, and effective colonization of the human host. In our final section, we summarize the evolution of meningococcal vaccines and their current state while emphasizing the value of ongoing molecular research into the pathogen's epidemiology and structural analysis of its antigens. IMD is a major global source of morbidity and mortality and a public health concern. IMD can manifest as an epidemic with breakouts or as an endemic illness with sporadic instances. There are 13 serogroups of Neisseria meningitis strains, however, only five (A, B, C, W-135, and Y) account for the majority of IMD globally. IMD poses a risk to people of all ages, although young children and teenagers are especially at risk. Meningitis and septicemia are the two clinical symptoms of IMD that occur most frequently, while both clinical presentations can occasionally exist. Age might affect the clinical pattern; in early childhood, the clinical manifestations could be more subtle, and the diagnosis may be trickier than in older kids or teenagers. In 4.3-11.2% of instances, there are sequelae, and death occurs in 6-10% of cases. Although vaccination remains the most effective method of preventing meningococcal disease, it is crucial to identify children with meningococcal infection as soon as possible to begin systemic antibiotic therapy. The prevalence of the disease has decreased as a result of the recent introduction of various meningococcal vaccinations on a global scale. Increasing meningococcal disease vaccination rates, keeping an eye on IMD, and creating a special vaccine that can protect against all of the major meningococcal strains should be the priorities for the upcoming few years.

## Introduction and background

Invasive meningococcal disease (IMD) is a major global health issue and the primary contributor to illness and death on a global scale. This condition is the result of infection by *Neisseria meningitidis*, an anaerobic gram-negative diplococcus bacterium. IMD affects approximately 500,000 people globally each year, with prevalence rates ranging from 1 per 100,000 individuals annually in North America and Europe to 10 per 100,000 in the sub-Saharan African region known as the "meningitis belt." Fatality occurs in 6-10% of cases, while sequelae impact 4.3-11.2% of those affected [1].

IMD caused by *N. meningitidis*, poses a significant threat of morbidity and mortality. Although newborns are most susceptible to IMD, there is also a heightened risk among adolescents and young adults. Worldwide, there are regional variations in the prevalence of meningococcal serogroups that cause meningitis and IMD. Meningococcal vaccinations have been developed to lessen the burden of IMD based on epidemiologic evidence. Since the development of a serogroup C conjugate vaccination, serogroup C IMD has significantly decreased in Europe. In Europe, serogroup B predominates, while serogroup Y IMD cases have been rising recently. Serogroup C and Y diseases have seen a decline in the United States due to the introduction of quadrivalent meningococcal conjugate vaccines (covering serogroups ACWY). However, serogroup B disease remains prevalent and is currently the leading cause of outbreak-related illness. In the African meningitis belt, the introduction of a conjugate vaccine for serogroup A has successfully reduced meningitis cases associated with that serogroup. Since 2006, outbreaks of the previously rare serogroup X disease have emerged in this region. Additionally, outbreaks of serogroup B IMD have been reported in both Europe and the United States, despite recent vaccine approvals by the European Medicines Agency and the U.S. Food and Drug Administration. Targeting adolescents and young adults for meningococcal vaccination campaigns is crucial to mitigate IMD-related morbidity and mortality, with the potential for positive herd effects on the broader community [2].

Meningococcal disease caused by *N. meningitidis* exhibits a substantial case fatality rate. The majority of these infections are attributed to 12 distinct serogroups, including A, B, C, W-135 (W), and Y. India's meningococcal disease burden and epidemiology are not well understood. To consolidate information on the prevalence of meningococcal disease, its epidemiology, and the recommended vaccine against it in India, we conducted a narrative review together with a methodical search [3]. Nearly 200 years have passed since meningococcal meningitis was recognized as a significant issue. The "meningitis belt" in Africa experiences recurring outbreaks of the disease during hot, dry weather, while East Africa, which lies outside of this belt, experiences epidemics more frequently in cold, dry months. In crowded settings like refugee camps and army barracks, the virus is mostly spread from person to person by nasopharyngeal carriers. Meningococcal meningitis is spread through a combination of factors, including rural-to-urban migration, the fundamental structural issues with housing in slums and squatter settlements, and an overburdened transportation network [4].

In Germany and other European nations, IMD is an illness that requires reporting. IMD prevention is highly relevant to public health despite the low prevalence of the disease in the population because of its high mortality and potential for long-term effects. This study's main objective is to outline important epidemiological and economic aspects of IMD in Germany to aid national decision-making procedures for putting improved prevention strategies into action [5].* N. meningitidis* (the meningococcus) causes enormous illness and mortality worldwide and can result from either epidemic outbreaks or sporadic cases of invasive infections. *N. meningitidis* epidemiology is dynamic and unpredictable. A rise in atypical clinical presentations appears to be linked to specific developing meningococcal genotypes. Early symptoms can indeed differ and are typically non-specific. Atypical clinical manifestations, such as abdominal presentations, septic arthritis, and bacteremic pneumonia, might cause misdiagnosis. Some of these are typically linked to increased case fatality rates due to delayed optimum care. Clinicians and public health professionals should be more knowledgeable about these uncommon but potentially serious presentations to make quick diagnoses and provide effective case management. In this research, we discussed atypical panels of meningococcal invasive disease clinical presentations that were related to recent changes in meningococcal epidemiology [6].

## Review

Methodologies

**Figure 1:**
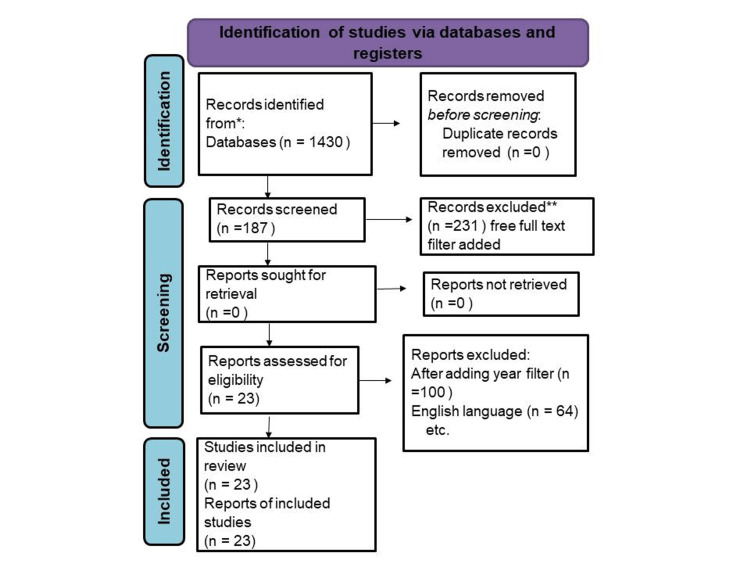
PRISMA flow diagram for an overview of meningococcal disease’s recent diagnostic and treatment model *Consider, if feasible to do so, reporting the number of records identified from each database; ** If ambulation tools were used, indicate how many records were excluded by a human and how many were excluded by automation tools.

We undertook a comprehensive search through PubMed and CENTRAL in August 2022 using keywords such as “An Overview Of Meningococcal Disease Recent Diagnostic And Treatment Model” and “Invasive meningococcal disease” (((An Overview Of Meningococcal Disease Recent Diagnostic And Treatment Model (Title/Abstract)) OR (An Overview Of Meningococcal Disease Recent Diagnostic And Treatment Model (Title/Abstract))) OR (“An Overview Of Meningococcal Disease Recent Diagnostic And Treatment Model” (MeSH Terms)) AND ((“Invasive meningococcal disease” (Title/Abstract)) OR (IMD (Title/Abstract))) OR (“Invasive meningococcal disease” (MeSH Terms)). We additionally searched for key references from bibliographies of the relevant studies. The article was added to this review between 1996 and 2022. One reviewer independently monitored the retrieved studies against the inclusion criteria, in the beginning, based on the title and abstract and then on full texts. Another reviewer also reviewed approximately 20% of these studies to validate the inclusion of studies. Differences were resolved through discussion. One reviewer extracted the data from studies according to the mental, physical, and social health aspects of IMD. Data were extracted and tabulated on details about the study design, population, overview of meningococcal disease's recent diagnostic and treatment model instrument used, IMD domain assessed, and study findings. We included studies that assessed the effect of an overview of meningococcal disease's recent diagnostic and treatment model both during and after treatment irrespective of the study design, geographic location, age, gender, or type of treatment. For inclusion, both published and unpublished studies in the English language were considered. We excluded studies that were published in other languages because of resource limitations or if the full-text articles were unavailable to reviewers. We also excluded studies in which an overview of meningococcal disease’s recent diagnostic and treatment model was not assessed, as shown in Figure 1.

Epidemiology

Infections like bacterial meningitis and other dangerous illnesses are frequently brought on by *N. meningitidis*. With significant variations in illness incidence and serogroup distribution, *N. meningitidis* has a very varied epidemiological profile. The majority of meningococcal illness cases globally are caused by six serogroups (serogroups A, B, C, W-135, X, and Y); each serogroup has a distinct epidemiological profile of the disease. For endemic illness brought on by serogroup B strains, there is no vaccination currently available. There are two licensed tetravalent (A/C/Y/W-135) meningococcal vaccines in the US: one is a polysaccharide-protein conjugate vaccination and the other is a pure polysaccharide product. All teenagers and other populations at high risk of illness should receive the conjugate vaccine, even though immunization rates are currently low in these groups. Infant immunization will be required for a comprehensive program to prevent invasive meningococcal infection in the United States; numerous conjugate vaccines for infants may become available shortly. Additionally, broad-spectrum vaccines are necessary for endemic serogroup B illnesses [7].

*N. meningitidis* stands as a prominent culprit behind bacterial meningitis and septicemia, contributing significantly to high case fatality rates and enduring consequences for survivors on a global scale. These bacteria encompass 12 recognized serogroups, with six (A, B, C, W, X, and Y) primarily responsible for IMD. The prevalence of IMD, as well as the serogroups causing it, exhibits substantial geographical and temporal variations. Fortunately, effective vaccinations targeting each of these serogroups are already available or soon will be. Over the past two decades, much of the world has witnessed a decline in IMD prevalence, owing to ongoing trends, successful meningococcal immunization initiatives, and various contributing factors. Meningococcal C conjugate vaccinations were launched in the early 2000s and were linked to rapid drops in meningococcal C disease, whereas meningococcal A conjugate vaccines were adopted across the African meningitis belt and were practically entirely responsible for meningococcal A disease elimination. As a result, IMD caused by various serogroups has increased in importance. Particularly, the advent of a meningococcal group W clone that is particularly virulent has prompted many nations to switch from monovalent meningococcal C through quadrivalent ACWY conjugate vaccines, which they have used in their national immunization programs. Furthermore, the most common strain causing pediatric IMD in Australia and Europe is now protected against by the recent approval of two protein-based, broad-spectrum meningococcal B vaccines. This paper discusses the prevalence of IMD worldwide, its historical trends across all continents, the IMD-causing serogroups, the effects of meningococcal vaccination campaigns, and the necessity to eradicate this deadly condition in the future [8].

Treatment

Clinical Feature for IMD

The initial stages of IMD can present with symptoms resembling a typical bacterial infection, posing challenges for physicians. Subsequently, specific symptoms of meningococcal infection including headache, neck stiffness, photophobia, and altered mental state, may precede the onset of a general febrile illness characterized by chills, muscle aches, nausea, and vomiting. However, it's important to note that less than one-third of patients will exhibit this "typical" combination of symptoms. Meningococcal septicemia, featuring signs of circulatory failure, shock, and the distinctive petechial or purpuric rash, can progress to sepsis in 40% to 70% of individuals with meningococcal disease [9].

**Figure 2:**
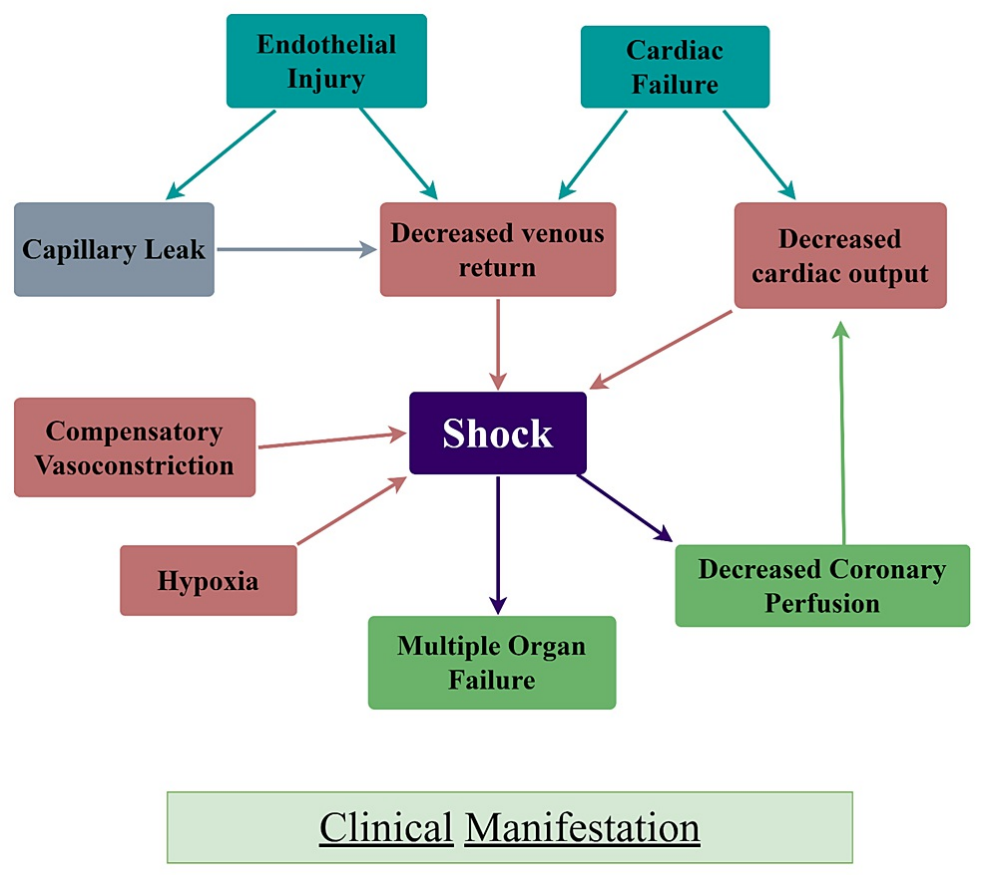
Clinical manifestation of meningococcal disease Credit: Image created by the author

Meningococcal disease's recognizable clinical signs don't manifest until rather late. By identifying early signs of sepsis, primary care providers may be able to identify more children and reduce the time it takes for hospital admission. It is important to emphasize the importance of parents and medical professionals recognizing these early symptoms when diagnosing meningococcal illness (Figure 2) [10].

Vaccines

Given the substantial risk of mortality and severe complications associated with the infection, even when treated with antibiotics in a timely manner, vaccination stands out as the most reliable approach to prevent meningococcal disease. The overall burden of the disease has decreased as a result of the recent introduction of new meningococcal vaccinations across the globe [1].

Meningitis and sepsis, two potentially fatal illnesses, are mostly brought on by *N. meningitidis*. There are currently vaccines available for five of the six serogroups that cause meningococcal illness as a result of breakthroughs in vaccine development (A, B, C, W, and Y). The prevalence of MenC illness and carriage has significantly decreased in Europe, largely attributable to successful vaccination campaigns employing monovalent meningococcal serogroup C (MenC) conjugate vaccines. Similarly, in the African meningitis belt, MenA illness has been nearly eliminated, thanks to the implementation of a monovalent MenA conjugate vaccination program. In response to the emergence of non-vaccine serogroups, recommendations in several countries have evolved gradually from monovalent conjugate vaccines to quadrivalent MenACWY conjugate vaccines, which provide broader protection against a wider range of serogroups. Wide-coverage, protein-based MenB vaccines have recently demonstrated real-world efficacy, which is encouraging. Meningococcal outbreaks can be controlled with the help of vaccines. Meningococcal illness remains a worldwide public health issue despite significant advancements. It is necessary to conduct further research on evolving epidemiology. The development of next-generation pentavalent vaccines, such as a MenACWYX conjugate vaccine and a MenACWY conjugate vaccine paired with MenB, is still ongoing and is anticipated to help eliminate meningitis globally [11]. Meningitis and septicemia are mainly brought on by meningococcus infection, which is a global problem. Meningococcus is typically found as a commensal dweller in the nasopharynx in about 10% of adults and may be present in over 25% of people throughout adolescence. Humans are the sole natural reservoir for these bacteria. Reduced mortality, which affects up to 10% of people with IMD, is only possible with early detection of meningococcal infection and intensive treatment. If treatment is given insufficiently or is given slowly, this number may be much higher. To help improve patient outcomes, early administration of potent parenteral antibiotic therapy as well as rapid identification and appropriate care of IMD consequences, such as circulatory shock and elevated intracranial pressure, are essential [12].

Serogroup A carriage dynamics

The epidemiological pattern of meningococcal disease is characterized by a region's coverage during the dry season followed by an endemic frequency during the rainy season. This pattern has made serogroup A the leading cause of meningitis in the African belt. Epidemics tend to occur in cycles spanning seven to ten years, typically in the second quarter of the dry season [13]. Meningococcal serogroup A was responsible for the majority of epidemics prior to the development of a conjugate vaccine against MenA, but serogroup C, and more recently, serogroups W and X, have also played a role. Compared to Europe, youngsters and young adults in this region are more likely to be carriers. Close family members, as opposed to other household contacts, tend to carry pathogenic serogroups at a higher rate, and individuals sharing a room with IMD patients have exhibited higher rates than other household members [14,15]. Higher case-carrier ratios suggest that the transition from epidemic to seasonal hyper-endemic periods is associated with an increased risk of meningitis given colonization, while epidemics are likely triggered by a substantial increase in transmission and colonization [16].

Serogroup C carriage dynamics

Comparatively, serogroup C carriage is less frequent when compared to other serogroups. Nevertheless, research findings in various settings have been inconsistent, with some studies reporting low carriage rates during MenC disease outbreaks, while others have found notably higher rates [17,18]. In the case of serogroup C's highly virulent strains, which are more prone to transmission, the brief duration of carriage results in a lower prevalence and an elevated risk of fatality. Consequently, the likelihood of severe disease is also heightened [16]. These strains typically lead to the development of meningitis several days after the initial meningococcal infection. These characteristics collectively contribute to the rapid dynamics of MenC infection, particularly in the case of the sequence type-11 clonal complex strains, which exhibit low carriage incidence and a higher risk of IMD [19,20].

Serogroup B

While vaccines are available for the three most common meningococcal capsular groups, an approved vaccine for epidemic capsular group B meningococcal disease has been lacking for decades. Recently, two new vaccines have gained approval for safeguarding against group B illness. Although these vaccines exhibit immunogenicity and are believed to have a satisfactory safety record, numerous scientific inquiries persist concerning their effectiveness against a range of endemic meningococcal strains, the duration of protection they provide, potential herd immunity effects, and whether evolving antigenic variations will diminish their efficacy over time. Furthermore, these vaccines present societal challenges in the United States, including the issue of high vaccine costs in the context of historically low incidence rates of meningococcal disease [21].

Vaccines in China

Due to the high incidence of meningitis in the period when vaccinations were available, *N. meningitidis*-caused meningococcal meningitis is a communicable disease in China. Following the introduction of the meningococcal vaccine in the 1980s, the disease's incidence significantly decreased. Currently, meningococcal meningitis vaccine formulations in polysaccharide, conjugate, and combination forms are available on the Chinese market; nearly all of these products are made locally. To increase the degree of meningococcus prevention and control, it is required to further improve national meningococcal surveillance. However, it is important to keep an eye on the immunological effectiveness and long-lasting protection of vaccines. More critically, more money has to be spent on serogroup B meningococcal vaccine development [22].

Global epidemiology of serogroup B

Meningococcal disease is a major global cause of meningitis and sepsis, characterized by a high case fatality rate and frequent long-term consequences. Fatal infections often result from *N. meningitidis* serogroups A, B, C, W, X, and Y, with unexpected epidemiological patterns that can trigger community epidemics, significantly impacting people's health, well-being, and work capacity. Serogroup B is currently the most prevalent in Latin America and the primary contributor to meningococcal disease in Europe and North America. Conjugate vaccines have effectively managed endemic and pandemic diseases linked to serogroups A, C, W, and Y, with more recent efforts involving geographically tailored outer membrane vesicle-based vaccines for serogroup B. A notable improvement to our arsenal against* N. meningitidis *is the addition of two novel protein-based vaccines because they offer comprehensive protection against a wide range of serogroup B and other group strains. These vaccines' early safety, efficacy, and impact results are encouraging. As they have the potential to save lives and avert grave consequences, these innovative serogroup B vaccinations should be actively evaluated for those who are more susceptible to the disease as well as for controlling serogroup B outbreaks that arise in institutions or certain locations. In-depth research into the needs of each country will be necessary for integration into national initiatives [23].

## Conclusions

IMD is a serious and potentially fatal issue for the global community. It is essential to diagnose meningococcal illness in children as soon as possible to provide systemic antibiotic therapy right away and prevent death or long-term consequences. The most effective method of preventing meningococcal illness is vaccination. The frequency of IMD has dramatically decreased recently in several countries; thanks to the advent of novel conjugate vaccinations that are efficient in very young children. A further expansion of IMD prevention options will be made possible by the advent of new MenB vaccinations. In the coming years, it should be a priority to boost meningococcal disease vaccination rates, keep an eye on IMD, and create a special vaccine that can protect against all of the major meningococcal strains.
